# Zinc Inhibits the GABA_A_R/ATPase during Postnatal Rat Development: The Role of Cysteine Residue

**DOI:** 10.3390/ijms24032764

**Published:** 2023-02-01

**Authors:** Sergey A. Menzikov, Danila M. Zaichenko, Aleksey A. Moskovtsev, Sergey G. Morozov, Aslan A. Kubatiev

**Affiliations:** Institute of General Pathology and Pathophysiology, 8, Baltiyskaya St., 125315 Moscow, Russia

**Keywords:** zinc, GABA_A_ receptor/ATPase, β subunits, cortical synaptoneurosomes, cysteine residue, postembryonal development

## Abstract

Zinc ions (Zn^2+^) are concentrated in various brain regions and can act as a neuromodulator, targeting a wide spectrum of postsynaptic receptors and enzymes. Zn^2+^ inhibits the GABA_A_Rs, and its potency is profoundly affected by the subunit composition and neuronal developmental stage. Although the extracellular amino acid residues of the receptor’s hetero-oligomeric structure are preferred for Zn^2+^ binding, there are intracellular sites that, in principle, could coordinate its potency. However, their role in modulating the receptor function during postembryonic development remains unclear. The GABA_A_R possesses an intracellular ATPase that enables the energy-dependent anion transport via a pore. Here, we propose a mechanistic and molecular basis for the inhibition of intracellular GABA_A_R/ATPase function by Zn^2+^ in neonatal and adult rats. The enzymes within the scope of GABA_A_R performance as Cl^−^ATPase and then as Cl^−^, HCO_3_^−^ATPase form during the first week of postnatal rat development. In addition, we have shown that the Cl^−^ATPase form belongs to the β1 subunit, whereas the β3 subunit preferably possesses the Cl^−^, HCO_3_^−^ATPase activity. We demonstrated that a Zn^2+^ with variable efficacy inhibits the GABA_A_R as well as the ATPase activities of immature or mature neurons. Using fluorescence recording in the cortical synaptoneurosomes (SNs), we showed a competitive association between Zn^2+^ and NEM in parallel changes both in the ATPase activity and the GABA_A_R-mediated Cl^−^ and HCO_3_^−^ fluxes. Finally, by site-directed mutagenesis, we identified in the M3 domain of β subunits the cysteine residue (C313) that is essential for the manifestation of Zn^2+^ potency.

## 1. Introduction

Neuronal plasticity is directly associated with alterations in the expression, properties, and function of plasma membrane channels and transporters that dissipate or generate ion gradients, respectively [1,2,3]. The divalent transition metal cation zinc (Zn^2+^) is concentrated in multiple brain regions, including the cerebral cortex, hippocampus, hypothalamus, and amygdala and seems to act as a neuromodulator, targeting a wide spectrum of postsynaptic receptors [4,5]. Specifically, Zn^2+^ modulates the sensitivity of inhibitory and excitatory receptors to their respective neurotransmitters [6,7,8].

As members of the pentameric ligand-gated ion channel (pLGICs) family, γ-aminobutyric acid type A receptors (GABA_A_Rs) are inhibited allosterically by micromolar concentrations of Zn^2+^ [9]. Recent structural studies have provided some insight into the mechanism of inhibition underlying GABA-induced responses to Zn^2+^ [10]. Once in the channel, Zn^2+^ may first bind to a histidine residue and then nullify the γ-aminobutyric acid (GABA) response by physically plugging the pore, thereby reducing the single Cl^−^ current. This residue is located at the extracellular end of the ion channel lining the TM2 domain of the β3 subunit and may form part of a zinc-binding site [11]. The Zn^2+^ inhibitory potency is profoundly affected by the subunit composition (αβ or αβγ) of GABA_A_Rs [12] and varies according to the developmental stage [13,14]. This is physiologically relevant because Zn^2+^ ions could be critically involved in various pathological processes (e.g., seizures and temporal-lobe epilepsy) where subunit expression may be altered [15,16,17]. In addition to the extracellular sites, there are intracellular amino acid residues in the receptor’s hetero-oligomeric structure that could, in principle, coordinate Zn^2+^ binding; however, their role in modulating the receptor remains unclear. The GABA_A_R β3 subunit possesses a zinc-sensitive ATPase that enables the energy-dependent transport of Cl^−^ via a receptor pore [18,19]. In addition, such a GABA_A_R-coupled ATPase is involved in seizures [20]. Given that Zn^2+^ ions are released both extra- and intracellularly [4,5], and ATPase is localized on the intracellular side of the channel [21], there is considerable interest in determining the molecular mechanisms through which Zn^2+^ modulates ATPase function. Furthermore, the molecular determinants that mediate the sensitivity of GABA_A_R to Zn^2+^ ions at different developmental stages are not fully understood.

Zinc is a signaling molecule involved in the regulation of various enzymes by inhibiting their catalytic activity [22]. Zn^2+^ at nano- or micromolar concentrations inhibits “metallobinding” proteins by interacting with an active center that contains catalytic dyads or triads of glutamate, histidine, and cysteine residues. Early studies have provided evidence of the sulfhydryl and disulfide groups’ involvement in GABA_A_R responses [23], notably two cysteine residues located at the external N-terminus and most of the extra cysteines (1–11) of the GABA_A_R subunits that can significantly contribute to redox modulation [24]. In addition, transmembrane domains can contain cysteines whose positions show a relatively high degree of conservation. Specifically, a single cysteine at the M3 domain is present in virtually all GABA_A_R subunit subtypes (including β subunits). Although the modulation of such cysteine residue (C313) by redox agents has been shown in homomeric GABA_A_R β3 subtypes [25], its sensitivity to zinc is unknown.

There is strong evidence showing that synaptic sulfhydryl groups of ionic channels and transporters are targets for electrophiles [26]. *N*-ethylmaleimide (NEM) is a membrane-permeant alkylating agent that modifies the thiol groups of cysteine residues via the formation of a covalent thioether bond. Specifically, NEM can cause an increase in the frequency of GABA_A_R-mediated postsynaptic currents [27,28] or eliminate the depolarization- or post-burst-induced suppression of GABA_A_R-mediated inhibition in CA1 pyramidal cells [28,29]. Moreover, NEM modulates the GABA_A_R-mediated Cl^−^ or HCO_3_^−^ fluxes in cortical neurons via regulating the desensitization/resensitization in a bicarbonate-dependent fashion [19]. However, the specific mechanism by which NEM functions to modulate GABA_A_Rs has yet to be established. Considering that NEM, in contrast to other thiol agents, causes a decrease in the neuronal concentration of ATP [19,30], we aimed to use NEM to test the hypothesis that cysteine residues contribute significantly to the Zn^2+^-mediated modulation of the ATPase activity.

Here, we propose a mechanistic and molecular basis for the inhibition of intracellular GABA_A_R/ATPase function by zinc in neonatal and adult rats. The enzymes within the scope of GABA_A_R performance as Cl^−^ATPase and then as Cl^−^, HCO_3_^−^ATPase form during the first week of postnatal rat development. The GABA_A_R β1 and β3 isoforms, in contrast to the β2 subunit, possess the ATPase activity that facilitates re-establishing the anion gradients into neurons. In addition, we have shown that the Cl^−^ATPase form belongs to the β1 subunit, whereas the β3 subunit preferably possesses the Cl^−^, HCO_3_^−^ATPase activity. We demonstrated that a Zn^2+^ with variable efficacy inhibits the GABA_A_R as well as the ATPase activities of immature or mature neurons. Using fluorescence recording in the cortical synaptoneurosomes (SNs), we showed a competitive association between Zn^2+^ and NEM in parallel changes both in the ATPase activity and the GABA_A_R-mediated Cl^−^ and HCO_3_^−^ fluxes. Finally, by site-directed mutagenesis, we identified in the M3 domain of β subunits the cysteine residue (C313) that is essential for the manifestation of Zn^2+^ potency.

## 2. Results

### 2.1. Zn^2+^ Inhibits the ATPase Activity in Neonatal and Adult Rats

**Figure 1:**
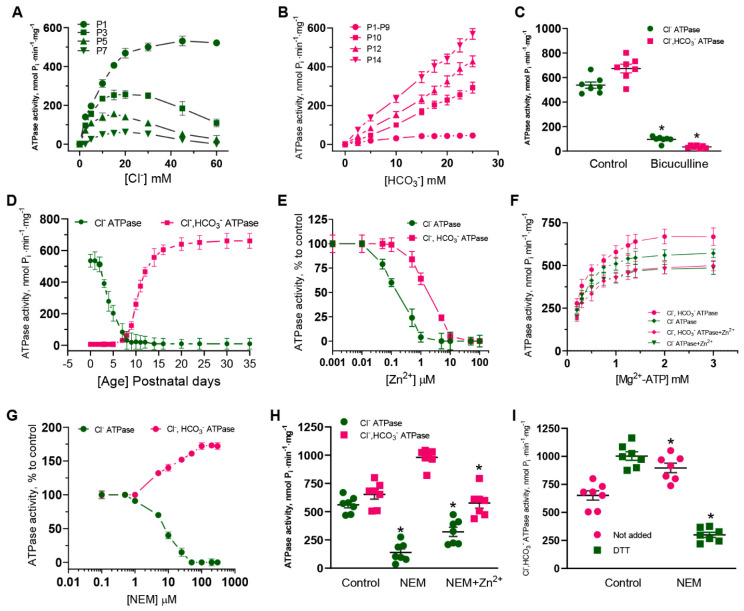
Zinc potency for ATPase activity is affected from brain development. (**A**) Cl^−^ concentration activation curves determined for ATPase of the plasma membranes performed from cerebral cortex during P1–P7 days (*n* = 7). (**B**) HCO_3_^−^ concentration activation curves measured for ATPase of the plasma membranes performed from cerebral cortex during P1–P14 days (*n* = 5). (**C**) Bar chart showing average maximum Cl^−^- and Cl^−^, HCO_3_^−^ATPase activities before and after 40 µM bicuculline application: Student’s unpaired *t*-test, *p* < 0.0001 (*n* = 7) and *p* < 0.0001 (*n* = 7). (**D**) Curves of Cl^−^- or Cl^−^, HCO_3_^−^ATPase activities determined in the plasma membranes performed from cerebral cortex during P1-P30 days (*n* = 7). (**E**) Zn^2+^ concentration inhibition curves for Cl^−^- or Cl^−^, HCO_3_^−^ATPase activities (*n* = 5). (**F**) Mg^2+^-ATP concentration activation curves determined for Cl^−^- or Cl^−^, HCO_3_^−^ATPase activities before and after 20 µM Zn^2+^ application. (**G**) NEM concentration modulation curves determined for Cl^−^- or Cl^−^, HCO_3_^−^ATPase activities. (**H**) Bar chart showing average maximum Cl^−^- or Cl^−^, HCO_3_^−^ATPase activities before or after 300 µM NEM and 300 µM NEM + 20 µM Zn^2+^ in an experimental medium: Student’s unpaired *t*-test, *p* < 0.0001 (*n* = 7), *p* < 0.0001 (*n* = 7), *p* = 0.4387 (*n* = 7) and *p* < 0.0001 (*n* = 7). (**I**) Bar chart showing average maximum Cl^−^, HCO_3_^−^ATPase activity before or after 300 µM NEM or 300 µM NEM + 2 mM DTT application: Student’s unpaired *t*-test, *p* < 0.0015 (*n* = 7) and *p* < 0.0001 (*n* = 7). Data are presented as mean ± SEM. * *p* < 0.05, ns, not significant.

To study the importance of ATPase in the early “ontogenetic switch” of GABAergic transmission [1,31,32], we assessed the effects of Cl^−^ and HCO_3_^−^ in ATPase activation during postnatal development (days P1–P30) in rats. The results revealed the essential role of Cl^−^ (30–60 mM) in the ATPase activity (537.8 ± 26.0 nmol P_i_·min^−1^·mg^−1^) at days P1 to P2, and to a lesser extent at days P5 to P7 (Figure 1A). While HCO_3_^−^ does not affect the ATPase activity during days P1–P9, it caused a significant increase in enzyme activity (569.3 ± 66.8 nmol P_i_·min^−1^·mg^−1^) at days P10 to P14 with a maximal effect in the concentration range of 20 to 30 mM (Figure 1B). Both the Cl^−^ATPase as well as the Cl^−^, HCO_3_^−^ATPase activity, in contrast to HCO_3_^−^ATPase (not shown), were inhibited by 40 µM bicuculline (Figure 1C). To determine whether there are two enzyme forms (Cl^−^ATPase and Cl^−^, HCO_3_^−^ATPase), we tested these enzyme activities at days P1 to P35. The Cl^−^ATPase activity appeared for the duration of time between days P1 to P7 with maximal effectiveness at days P1 to P3. While the Cl^−^, HCO_3_^−^ATPase was observed after day P8, the maximal activity (673.4 ± 35.5 nmol P_i_·min^−1^·mg^−1^) occurred at day P15 of postnatal development and older (Figure 1D). These results are consistent with observations showing that [HCO_3_^−^]_i_ homeostasis is provided by the plasma membrane transporters (primary, NCBE, and carbohydrase) around postnatal day 12 [2,32,33]. By way of comparison, we tested the effect of Zn^2+^ on the Cl^−^ATPase or Cl^−^, HCO_3_^−^ATPase activities of the plasma membranes from the brains of neonatal (P1) and adult (P35) rats. The Zn^2+^ at concentrations ranging from 0 to 100 µM completely blocked these enzyme activities, with a maximum effect at 1 µM (I_50_ = 0.2 µM) and 10 µM (I_50_ = 2.0 µM), respectively (Figure 1E). As shown in Figure 1F, the inhibiting effect of Zn^2+^ on the studied ATPase activity was dependent on the concentration of Mg^2+^-ATP (0.3–3 mM) in the incubation medium.

NEM (200–400 µM) can both activate as well as inhibit GABA_A_R efficacy [28,29]. To clarify the role of cysteine residues, we explored the effect of NEM on the Cl^−^ATPase and the Cl^−^, HCO_3_^−^ATPase. As illustrated in Figure 1G, NEM at concentrations ranging from 1 to 100 µM completely blocked the Cl^−^ATPase activity in neonate (P1) rats. Contrariwise, the Cl^−^, HCO_3_^−^ATPase activity was elevated approximately two-fold in the presence of NEM (200–400 µM). The inhibition of Cl^−^ATPase activity did not significantly recover in the presence of 20 µM Zn^2+^, whereas the NEM activation of the Cl^−^, HCO_3_^−^ATPase was eliminated by Zn^2+^, suggesting a similar binding site (Figure 1H). In addition, the activating effect of 300 µM NEM was eliminated by dithiothreitol (DTT) at 2 mM, indicating the involvement of the thiol groups of cysteine residues (Figure 1I). Meanwhile, DTT activated the Cl^−^, HCO_3_^−^ATPase activity in the control animals (without NEM) this did not appear in the presence of zinc. These data concur with the literature. In particular, DTT has enhanced the responses of functional recombinant GABA_A_Rs by a mechanism in addition to Zn^2+^ chelation [25].

### 2.2. Zn^2+^ Inhibits the GABA_A_R-Mediated Cl^−^ Flux in Newborn and Mature Neurons

Neuronal intracellular chloride homeostasis is determined primarily by second-active cation-chloride co-transporters (CCCs) and by various ion channels (Cl^−^-channels activated in response to membrane-potential changes, intracellular Ca^2+^-channels, and pH-sensitive Cl^−^-channels) [33]. Considering that CCCs are sensitive to Zn^2+^ and NEM, to eliminate their possible influence on the GABA_A_R-mediated [Cl^−^]_i_ changes, the loop diuretic bumetanide was added to the experimental medium (50 µM) to act as a specific blocker of the Na^+^-(K^+^)-Cl^−^-cotransporter (NKCC1) and the K^+^-Cl^−^-cotransporter (KCC2) [34]. In immature neurons, the intracellular concentration of Cl^−^ ([Cl^−^]_i_) is approximately 30 mM [2], and the neuronal equilibrium potential for chloride (E_Cl_^−^) is positive relative to the resting membrane potential (V_m_); thus, the GABA_A_R activation results in a net passive Cl^−^ efflux and neuronal depolarization [35].

**Figure 2:**
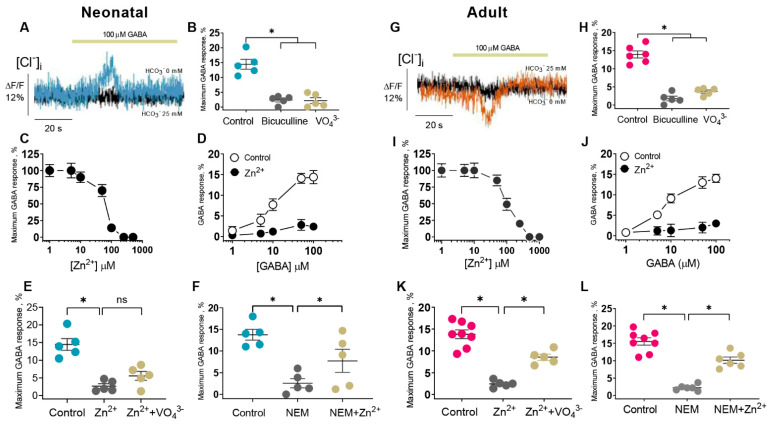
Zinc potency for GABA_A_R-mediated Cl^−^ flux is affected from neuron development stages. (**A**,**G**) Corresponding MQAE fluorescence changes for SNs (P1) and (P35) recording in response to 100 µM GABA application without or with 25 mM HCO_3_^−^ in an experimental medium, respectively. (**B**) Bar chart showing average maximum GABA response before and after 40 µM bicuculline or 25 µM VO_4_^3−^ application: Student’s unpaired *t*-test, *p* < 0.0001 (*n* = 5) and *p* < 0.0002 (*n* = 5). (**C**,**I**) Zn^2+^ concentration inhibition curve recording for average maximum the GABA response. (**D**,**J**) GABA concentration activation curve recording for MQAE fluorescence changes before and after 100 µM Zn^2+^ (*n* = 4). (**E**) Bar chart showing average maximum GABA response before and after 100 µM Zn^2+^ or 100 µM Zn^2+^ + 25 µM VO_4_^3−^ application: Student’s unpaired *t*-test, *p* < 0.0002 (*n* = 5) and *p* < 0.0062 (*n* = 5). (**F**) Bar chart showing average maximum GABA response before and after 300 µM NEM or 300 µM NEM + 100 µM Zn^2+^ application: Student’s unpaired *t*-test, *p* < 0.0001 (*n* = 5) and *p* = 0.1094 (*n* = 5). (**H**) Bar chart showing average maximum GABA response before and after 40 µM bicuculline or 25 µM VO_4_^3^ application: Student’s unpaired *t*-test, *p* < 0.0001 (*n* = 6) and *p* < 0.0400 (*n* = 5). (**K**) Bar chart showing average maximum GABA response before and after 200 µM Zn^2+^ or 200 µM Zn^2+^ + 25 µM VO_4_^3−^ application: Student’s unpaired *t*-test, *p* < 0.0001 (*n* = 8) and *p* < 0.0001 (*n* = 5). (**L**) Bar chart showing average maximum GABA response before and after 300 µM NEM or 300 µM NEM + 200 µM Zn^2+^ application: Student’s unpaired *t*-test, *p* < 0.0001 (*n* = 8) and *p* < 0.0001 (*n* = 5). Data are presented as mean ± SEM. * *p* < 0.05, ns, not significant.

The results show that the chloride-sensitive fluorescent quenching dye MQAE (n-(ethoxycarbonylmethyl)-6-methoxyquinolinium bromide) is a useful tool for determining intracellular chloride activity, and for the quantitative determination of chloride fluxes in neurons [36]. Using MQAE we measured the [Cl^−^]_i_ in the SNs (P1), which was approximately 28 mM. To explore the GABA_A_R activity, the SNs were initially loaded with MQAE and then exposed to a mediator. Previously, it was shown that GABA (1–100 µM) increased the Cl^−^, HCO_3_^−^ATPase activity and GABA_A_R-mediated Cl^−^ transport in the HEK 293FT and neuronal cells with a maximum effect at 100 µM [7,18]. As shown in Figure 2A, SNs (P1) demonstrated a fast (10 s) Cl^−^ efflux in response to the application of 100 µM GABA in HCO_3_^−^ free experimental medium with a maximum peak in fluorescence changes of 14.4 ± 1.6% followed by a reduction in MQAE fluorescence. However, in the presence of 25 mM HCO_3_^−^ in the experimental medium, the GABA-mediated Cl^−^ efflux did not appear. The addition of 40 µM of bicuculline or 25 µM of VO_4_^3−^ to the medium led to a suppression of GABA-mediated fluorescence changes (Figure 2B), indicating that Cl^−^ efflux from SNs (P1) is an ATP-dependent process via the GABA_A_R pathway [19]. Early research suggests that the inhibition of the GABA_A_R-mediated Cl^−^ current in the hippocampus by 50 µM Zn^2+^ was stronger in neonate rats (29.3%) than in adults (13%) [13]. In our study, Zn^2+^ in the range of 1 to 300 µM inhibited peak fluorescence changes at IC_50_ = 80 µM, with the maximum effect (100%) at a concentration of 200 µM (Figure 2C). The GABA concentration response curve (EC_50_ = 9.0 µM) revealed that the inhibition of GABA_A_R-mediated fluorescence changes by Zn^2+^ (100 µM) is non-competitive with little dependence on the mediator concentration (EC_50_ = 12.0 µM) (Figure 2D). In the presence of 25 µM VO_4_^3−^, the GABA-mediated response was slightly restored (not statistically significant) (Figure 2E). To clarify the role of cysteine residues in the Zn^2+^ modulating effect and considering the data in Figure 1G, we used NEM at 300 µM. As shown in Figure 2F, NEM completely eliminated the GABA-mediated Cl^−^ efflux from the neurons. In the presence of 100 µM Zn^2+^, the NEM effect was noticeably restored. In order to test whether a high concentration of GABA affects ligand modulation [37,38], we applied a low concentration of mediator. Specifically, 10 μM GABA induced a Cl^−^ efflux from the neurons and Zn^2+^ (100 µM) or NEM (300 µM) completely eliminated the effect of the mediator, confirming the absence of changes in the pharmacological properties of the receptor.

In mature neurons isolated from adult rats, the [Cl^−^]_i_ is approximately 6 mM [2], E_Cl_^−^ is negative, and GABA_A_R activation triggers a Cl^−^ influx and subsequent hyperpolarization [32]. We measured the [Cl^−^]_i_ in the SNs (P35) to be approximately 5 mM. To clarify the role of the GABA_A_R activity, the SN_S_ were initially loaded with chloride-sensitive dye and then exposed to the mediator. As shown in Figure 2G, SNs (P35) demonstrated a fast (10 s) Cl^−^ efflux in response to the application of 100 µM GABA in HCO_3_^−^ free experimental medium with a maximum peak in fluorescence changes of 14.0 ± 1.0%. The application of mediator induced a non-significant Cl^−^ influx into the SNs (P35) in the HCO_3_-free medium with a maximum peak in fluorescence changes of 6.0 ± 0.5% (Figure 2G). The added 40 µM bicuculline or 25 µM VO_4_^3−^ in the medium led to the suppression of the GABA-mediated fluorescence changes, confirming that Cl^−^ influx is an ATP-dependent process via a receptor pore (Figure 2H). Zn^2+^ in the range of 1 to 1000 µM inhibited the peak in fluorescence changes at IC_50_ = 100 µM, with maximum effect at a concentration of 500 µM (Figure 2I). The GABA concentration response curve (EC_50_ = 7.0 µM) revealed that inhibition of GABA_A_R-mediated fluorescence changes by 200 µM Zn^2+^ is non-competitive (EC_50_ = 7.3 µM) (Figure 2J). In the presence of 25 µM VO_4_^3−^, the GABA-mediated response was partially recovered, indicating close binding sites in the ATP-hydrolyzing center (Figure 2K). To establish the role of cysteine residues in the Zn^2+^ modulation effect, we used NEM as a drug-specific modulator of the ATPase activity. As shown in Figure 2L, NEM (300 µM) eliminated the GABA_A_R-mediated Cl^−^ influx from the neurons. In the presence of 200 µM Zn^2+^, the NEM effect was significantly restored.

### 2.3. Zn^2+^ Inhibits the GABA_A_R-Mediated HCO_3_^−^ Flux in Mature Neurons

**Figure 3:**
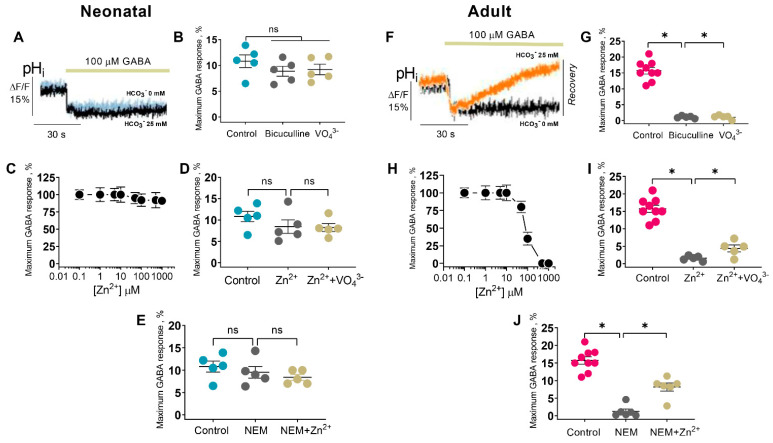
Zinc inhibits the GABA_A_R-mediated [HCO_3_^−^]_i_ recovery in mature neurons. (**A**,**F**) Corresponding BCECF fluorescence changes for SNs (P1) and (P35) recording in response to 100 µM GABA application without or with 25 mM HCO_3_^−^ in an experimental medium, respectively. (**B**) Bar chart showing average maximum GABA response before and after 40 µM bicuculline or 25 µM VO_4_^3−^ application: Student’s unpaired *t*-test, *p* = 0.2433 (*n* = 5) and *p* = 0.3434 (*n* = 5). (**C**,**H**) Zn^2+^ concentration inhibition curve recording for average maximum the GABA response. (**D**) Bar chart showing average maximum GABA response before and after 300 µM Zn^2+^ or 300 µM Zn^2+^ + 25 µM VO_4_^3−^ application: Student’s unpaired *t*-test, *p* = 0.2734 (*n* = 5) and *p* = 0.8019 (*n* = 5). (**E**) Bar chart showing average maximum GABA response before and after 300 µM NEM or 300 µM NEM + 300 µM Zn^2+^ application: Student’s unpaired *t*-test, *p* = 0.4974 (*n* = 5) and *p* = 0.4636 (*n* = 5). (**G**) Bar chart showing average maximum GABA response before and after 40 µM bicuculline or 25 µM VO_4_^3^ application: Student’s unpaired *t*-test, *p* < 0.0001 (*n* = 9) and *p* < 0.0001 (*n* = 5). (**I**) Bar chart showing average maximum GABA response before and after 300 µM Zn^2+^ or 300 µM Zn^2+^ + 25 µM VO_4_^3−^ application: Student’s unpaired *t*-test, *p* < 0.0001 (*n* = 9) and *p* < 0.0165 (*n* = 5). (**J**) Bar chart showing average maximum GABA response before and after 300 µM NEM or 300 µM NEM + 300 µM Zn^2+^ application: Student’s unpaired *t*-test, *p* < 0.0011 (*n* = 6) and *p* < 0.0004 (*n* = 6). Data are presented as mean ± SEM. * *p* < 0.05, ns, not significant.

The CO_2_ production and conversion in the intracellular HCO_3_^−^ has an outwardly directed flow after prolonged GABA exposure, despite a lower permeability via the receptor pore, with a consequent dramatic recovery in [HCO_3_^−^]_i_ [39,40]. pH-sensitive fluorescent dyes have been widely applied to monitor changes in intracellular pH. Among them, 2′,7′-bis-(2-carboxyethyl)-5-(and-6)-carboxyfluorescein (BCECF AM) is cell-permeable. With an increase in the pH of the cells, an increase in fluorescence is observed, and with a decrease in pH, the fluorescence is quenched [41]. To clarify the role of the GABA_A_Rs in the bicarbonate transport, the SNs were initially loaded with pH-sensitive dye (BCECF) and then exposed to GABA. Since the extracellular concentration of bicarbonate is about 25 mM [42], we studied the GABA_A_R-mediated HCO_3_^−^ efflux in the absence or presence of 25 mM HCO_3_^−^ in the experimental medium. The SNs (P1) in the absence or presence of 25 mM HCO_3_^−^ showed a rapid decrease in intracellular pH (pH_i_) in response to the GABA (100 µM) addition, with a maximum peak in fluorescence changes of approximately 10.6 ± 1.9% followed by a plateau (Figure 3A). Neither bicuculline at 40 µM or 25 µM VO_4_^3−^ affected the GABA-mediated HCO_3_^−^ efflux in the absence or presence of 25 mM HCO_3_^−^ (Figure 3B), indicating that it was not via a receptor-dependent pathway and was without the involvement of the ATPase system. Zn^2+^ in the range of 1 to 1000 µM did not affect the peak in fluorescence changes (Figure 3C). As shown in Figure 3D, in the presence of 25 µM VO_4_^3^, the GABA-mediated response was unchanged. Similarly, NEM (300 µM) with or without 300 µM Zn^2^ in the experimental medium did not change the fluorescence peak (Figure 3E).

To explore the GABA_A_R activity in adult rats, the SN_S_ (P35) were initially loaded with BCECF and then exposed to GABA. The SNs (P35) in the HCO_3_^−^-free medium showed a rapid pH_i_ decrease in response to the GABA (100 µM) addition with a maximum peak in fluorescence changes of 7.3 ± 1.0%, while in the presence of 25 mM HCO_3_^−^, the GABA-mediated HCO_3_^−^ efflux was elevated twice with a maximum peak in fluorescence change of 15.7 ± 1.0% and a subsequent rapid (30 s) recovery of [HCO_3_^−^]_i_ (Figure 3F). As illustrated in Figure 3G, 40 µM bicuculline, as well as 25 µM VO_4_^3−^, eliminated the recovery of GABA-mediated pH_i_ changes in the presence of 25 mM HCO_3_^−^, confirming that it is an energy-dependent process within the channel [19]. Zn^2+^ in the range of 1–1000 µM inhibits the recovery peak in fluorescence changes at IC_50_ = 90 µM and is completely eliminated at 400 µM (Figure 3H). As shown in Figure 3I, in the presence of 25 µM VO_4_^3−^, Zn^2+^-induced inhibition at 300 µM was partially restored, indicating close site binding in the ATP-hydrolyzing center. NEM (300 µM) completely eliminated the GABA-mediated [HCO_3_^−^]_i_ changes, but in the presence of 300 µM Zn^2+^, the NEM effect was noticeably restored (Figure 3J).

### 2.4. β3 Subunit Is Responsible for Zn^2+^-Sensitive [HCO_3_^−^]_i_ Recovery

**Figure 4:**
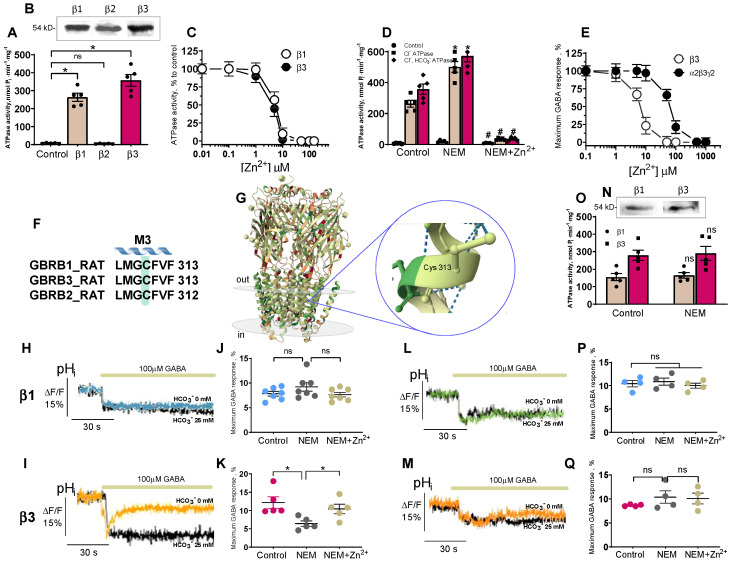
Zinc inhibits ATP-dependent recovery of [HCO_3_^−^]_i_ by β3 subunit. (**A**) Bar chart showing the ATPase activity in VLPs after expression in HEK 293FT cells of GABA_A_R β1, β2 or β3 subunit (*n* = 5). (**B**) Western blot analysis binding of VLPs with antibody against GABA_A_R β1, β2 or β3 subunit after expression of DNA GABA_A_R β1, β2 or β3 subunit in HEK 293FT cells. (**C**) Zn^2+^ concentration inhibition curves for ATPase activities in VLPs after expression in HEK 293FT cells of DNA GABA_A_R β1 or β3 subunit. (**D**) Bar chart showing the Cl^−^, HCO_3_^−^-ATPase activity in VLPs after expression in HEK 293FT cells of DNA GABA_A_R β1 or β3 subunit without or with 300 µM NEM or 300 µM NEM + 20 µM Zn^2+^ in an experimental medium: Student’s unpaired *t*-test, *p* < 0.0008 (*n* = 5), *p* < 0.0073 (*n* = 5) and *p* < 0.0001 (*n* = 5), *p* < 0.0001 (*n* = 5). (**E**) Zn^2+^ concentration inhibition curve recording for average maximum the GABA response recording in HEK 293FT cells expressing GABA_A_R β3 or α2β3γ2 subunits. (**F**) The amino acid sequences of β1, β2 and β3 subunits that contained the cysteine residues (Protein Bank). (**G**) Architecture of GABA_A_R-β3_cryst_ (five subunits from five are shown) and cysteine residue (C313) in M3 domain of β3 subunit (Protein Data Bank). (**H**,**I**) Corresponding BCECF fluorescence changes for HEK 293FT cells expressing GABA_A_R β1 or β3 subunit recorded in response to 100 µM GABA application without or with 25 mM HCO_3_^−^ in an experimental medium (*n* = 5). (**J**,**K**) Bar chart showing average maximum GABA response in HEK 293FT cells expressing GABA_A_R β1 or β3 subunit in the presence of 25 mM HCO_3_^−^ in an experimental medium, before and after 300 µM NEM or 300 µM NEM + 20 µM Zn^2+^ application: Student’s unpaired *t*-test, *p* = 0.1894 (*n* = 7) and *p* = 0.1453 (*n* = 7). (**L**,**M**) Corresponding BCECF fluorescence changes for HEK 293FT cells expressing GABA_A_R β1 or β3 subunit recorded in response to 100 µM GABA application without or with 25 mM HCO_3_^−^ in an experimental medium (*n* = 5). (**N**) Western blot analysis binding of VLPs with antibody against GABA_A_R β1 or β3 subunit after expression of mutant (C313A) GABA_A_R β1 or β3 subunit in HEK 293FT cells. (**O**) Bar chart showing ATPase activity in VLPs after expression in HEK 293FT cells of mutant (C313A) DNA GABA_A_R β1 or β3 subunit before and after 300 µM NEM application: Student’s unpaired *t*-test, *p* = 0.7121 (*n* = 5) and *p* = 0.8232 (*n* = 5). (**P**,**Q**) Bar chart showing average maximum GABA response in HEK 293FT cells expressing mutant GABAAR β1 or β3 subunit in the presence of 25 mM HCO_3_^−^ in an experimental medium, before and after 300 µM NEM or 300 µM NEM + 20 µM Zn^2+^ application: Student’s unpaired *t*-test, *p* = 0.2826 (*n* = 4) and *p* = 0.6583 (*n* = 4) and *p* = 0.2353 (*n* = 4) and *p* = 0.8668 (*n* = 4). Data are presented as mean ± SEM. * *p* < 0.05, ns, not significant; # *p* < 0.05, is significant between NEM and NEM + Zn^2+^.

Although, the structures of synaptic GABA_A_Rs are composed of 2α, 2β, and γ subunits, the functional and pharmacological properties are largely ensured by β subunits, of which the β3 subunit has an important role to play [43]. Previous studies of recombinant GABA_A_Rs report that in these three subunits (α2, β3, and γ2) only the β3 subunit possesses the Cl^−^, HCO_3_^−^ATPase activity [18]. To determine whether such ATPase characteristics are unique to β3 or whether β1 and β2 subunits could also manifest an enzyme activity that will be inhibited by Zn^2+^ [18], we expressed single rat β1, β2, or β3 subunits. The homomeric GABA_A_R β1 and β3 isoforms demonstrated Cl^−^ATPase or Cl^−^, HCO_3_^−^ATPase activities (263.6 ± 22.0 and 357.6 ± 32.7 nmol P_i_·min^−1^·mg^−1^, respectively), but did not appear in cells expressing the β2 subunit (Figure 4A). HEK 293FT cells expressing the GABA_A_R β1, β2, or β3 isoforms showed one band in the VLPs, with a molecular weight of approximately ~54 kDa, that bound to the antibodies against the GABA_A_R β1, β2, or β3 subunits, respectively (Figure 4B). As shown in Figure 4C, Zn^2+^ (1–100 µM) completely suppressed the enzyme activity of the GABA_A_R β1 and β3 isoforms. The addition of NEM (300 µM) in the experimental medium resulted in an increase in ATPase activities by approximately one and eight-tenths. In the presence of 20 µM Zn^2+^, the activating effect of NEM did not appear (Figure 4D).

Several recombinant receptor studies demonstrated that the potency of Zn^2+^ is higher on the αβ subtypes than the αβγ receptors [7,10,11]. Furthermore, the homomeric channels formed by only α2 or β2 subunits were non-competitively blocked by 10 µM Zn^2+^ to approximately the same extent (>80%) as the α1β2 isoform [44]. To ensure that the homomeric GABA_A_R subtypes were functional and would be inhibited by Zn^2+^, we examined the whole-cell flow evoked by GABA (100 µM) in HEK 293FT cells transfected with plasmid vectors containing GABA_A_R subunit cDNAs to produce the β1 or β3 subtypes. Considering that the homomeric β3 GABA_A_R isoforms demonstrated a marginal effect on the GABA-mediated Cl^−^ inflow in cells [18], in the present study, we used the more effective benzamidine (5 mM) instead of GABA [45]. By way of comparison, we tested the effect of Zn^2+^ on the homomeric β3 and the heteromeric α2β3γ2 GABA_A_R isoforms.

As shown in Figure 4E, Zn^2+^ at concentrations ranging from 0 to 500 µM completely blocked the MQAE fluorescence changes with maximum effects at 30 µM and 300 µM, respectively. While in the homomeric GABA_A_R ensembles containing the homomeric β1 or β3 isoforms, GABA at 100 µM induced a similar decrease in the pH_i_ of cells previously resuspended in a medium with or without 25 mM HCO_3_^−^ by approximately 8.0 ± 0.7% (Figure 4H). The HEK 293FT cells expressing the GABA_A_R β3 subtype in the HCO_3_^−^-free medium also manifested only a GABA-mediated decrease in pH_i_ (7.9 ± 0.9%) while in the presence of 25 mM HCO_3_^−^, the cells expressing the GABA_A_R β3 subtype in contrast to the β1 isoform, showed the GABA-mediated decrease in pH_i_ with a maximum peak fluorescence change of 14.0 ± 1.3%, which was recovered over 30 s (9.9 ± 0.5%) (Figure 4I). As shown in Figure 4J, NEM (300 µM) had no significant effect on the GABA-mediated HCO_3_^−^ flow in the cells expressing the homomeric GABA_A_R β1 isoform in the absence or presence of 20 µM Zn^2+^. While in the cells expressing the GABA_A_R β3 isoform, the NEM inhibiting effect was partially recovered by 20 µM Zn^2+^ (Figure 4K).

A single cysteine residue (C313) in the M3 domain is conserved in all GABA_A_R α, β, and γ subunits (Figure 4F). To test whether this cysteine residue formed, at least partially, the molecular basis for NEM modulation, this residue was mutated to alanine in the β1 and β3 subunits (C313A). The HEK 293FT cells expressing the mutant GABA_A_R β1 or β3 isoforms showed one band in the VLPs with a molecular weight of approximately ~54 kDa, that bound to the antibodies against the GABA_A_R β1 or the β3 subunit, respectively (Figure 4N). As shown in Figure 4O, the mutant (C313A) GABA_A_R β1 and β3 isoforms demonstrated Cl^−^ and Cl^−^, HCO_3_^−^ATPase activities of 218.8 ± 24.8 and 279.2 ± 29.0 nmol^−1^·P_i_·min^−1^·mg^−1^, respectively. The addition of 300 µM NEM in the experimental medium did not change the enzyme activities. The homomeric mutant GABA_A_R β1 or β3 (C313A) isoform still displayed the GABA-mediated pH_i_ drop in either the absence or presence of HCO_3_^−^ by approximately 8.0 ± 0.9% (Figure 4L,M), and this did not change after the application of NEM at 300 µM in the absence or presence of 20 µM Zn^2+^ (Figure 4P,R).

## 3. Discussion

Currently, the properties of GABA_A_Rs are studied using electrophysiological (e.g., patch-clamp) and non-electrophysiological (e.g., fluorescence-based) methods. Although the fluorescence-based, in contrast to the patch-clamp method, does not directly measure ionic current and ion-concentration-dependent changes of fluorescence signals as a result of ionic flux, using environmentally sensitive dyes may allow to detect the conformational changes [46]. We used fluorescence recordings to directly assess the contribution of anions on GABA_A_R functional activity during ontogenesis. Our results shed light on the substantial role of HCO_3_^−^ in the GABA efficacy in SNs isolated from adult rats (P35). In addition, we focused on the role of ATPase in the rapid re-establishment of anionic gradients after GABA_A_R responses. During the first days (P1–P4), Cl^−^, but not HCO_3_^−^, played a dominant role in the ATPase activity, while after postnatal day 10, a dominant role for HCO_3_^−^ and a minor role for Cl^−^ became apparent (Figure 1). The dramatic transient switching in the effectiveness and performance of the enzyme forms from Cl^−^ATPase to Cl^−^, HCO_3_^−^ATPase during early postnatal development demonstrates the involvement of primary-active transport in the recovery not only of [Cl^−^]_i_, but also [HCO_3_^−^]_i_ after day P10. In addition, it was established that Cl^−^ATPase is related to the GABA_A_R β1 isoform, whereas the Cl^−^, HCO_3_^−^ATPase belonged to the GABA_A_R β3 isoform. Most likely, this is associated with both the distinct properties of the subunits and their traffic changes to the cell surface [47]. Data from the literature shows the variable expression of mRNA GABA_A_R β1, β2, or β3 subunits in hippocampal granule cells during postnatal development. Specifically, in neurons isolated in samples between days P5 and P7, there is a significant expression of the GABA_A_R β1 subunit mRNA in contrast to the β2 or β3 subunits [14], and neurons isolated in samples from postnatal days 17–21 had an increased expression of the GABA_A_R β2 and β3 subunits’ mRNA. In this study, we show for the first time that the homomeric β3 isoform, in contrast to the β1 isoform, manifests an essential GABA-mediated HCO_3_^−^ outflow and its recovery. These data suggest the high probability of the presence of a distinctive mechanism for HCO_3_^−^ transport via the GABA_A_Rs. Notably, previous work has gained attention in the concluding observations that, out of the three β subunits, only the expression of β3 in the β1-β2 subunit knockout can fully maintain or restore inhibitory responses to control levels in the hippocampus [48].

**Figure 5:**
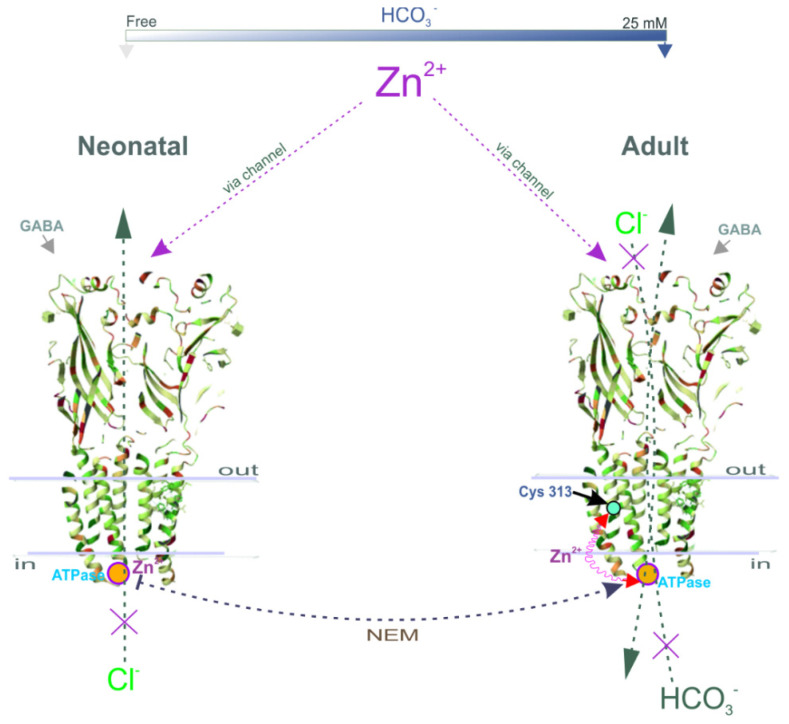
A hypothetical molecular mechanism for zinc inhibition of the GABA_A_R/ATPase function during neuronal development: (**left**) In neonatal neurons, Zn^2+^ similar as NEM forms the inactive complex with ATPase center and inhibited the GABA_A_R-mediated Cl^−^ efflux. (**right**) In mature neurons, Zn^2+^ inhibits the GABA_A_R-mediated Cl^−^ efflux. Modification of the Cys 313 thiol group by NEM activates the ATPase activity. Zinc eliminates the NEM effect, indicating their allosteric interaction. Architecture of GABA_A_R-β3_cryst_ (two subunits from five are shown) and cysteine residue (C313) in M3 domain of β3 subunit (Protein Data Bank).

Although early studies reported that Zn^2+^ inhibits both primary active transporters and secondary active cation-chloride cotransporters (CCCs), there are essential differences in their sensitivity to its inhibiting action. Specifically, Zn^2+^ inhibits the erythrocyte Ca^2+^ ATPase with a K_i_ of 80 pM [49], whereas CCCs are inhibited at high concentrations (I_50_ = 50 µM) [50]. In the present study, we propose a mechanistic and molecular basis for the inhibition of GABA_A_R-coupled ATPase by Zn^2+^ and its dependence on the stage of postembryonal development. The ATPase activity was inhibited by Zn^2+^, but its sensitivity to cations differed in neonatal (IC_50_ = 0.2 µM) and adult rats (IC_50_ = 2.0 µM), which is similar to the results of electrophysiological studies. Specifically, the GABA_A_R-mediated Cl^−^ current recorded in hippocampal slices isolated from postnatal rats (P1–P5) was inhibited by 80% at 100 µM Zn^2+^, in contrast to adult rats (30% inhibition at 400 µM Zn^2+^) [13,14]. In confirmation of these data, the GABA_A_R-mediated Cl^−^ flow in the SNs (P1) was inhibited by ~90% at 100 µM Zn^2 +^(I_50_ = 80 µM), in contrast to adult rats (80% inhibition at 250 µM Zn^2+^) (I_50_ = 100 µM) (Figure 2). Based on the received data, the physiological role of zinc in the modulation of GABA_A_R/ATPase functional activity is in question because zinc ions are released both extra- and intracellularly [16]. Cellular “free” zinc concentrations are between 10 and 1000 pM and these concentrations are similar to the affinity of Zn^2+^ for cytosolic “metallobinding” enzymes [22]. Effective micromolar zinc-inhibition can have physiological significance only at an extracellular binding with the receptor complex. However, some studies have shown that Zn^2+^ can penetrate via the channel as a complex ion with permeating anions resulting in the intracellular or membrane inhibition effect [51,52]. Specifically, on cultured hippocampal neurons it was showed that a continuous background release of GABA induced a standing-sensitive inward Cl^−^-current that was inhibited by bicuculline [51]. This leakage current is initially reduced in amplitude by 300 µM Zn^2+^ and eventually converted, in the continued presence of zinc, into discrete discontinuous transients appearing. It can be assumed that in our study we have also observed both the extra- and intracellular effects of Zn^2+^ on the GABA_A_R-mediated anion transport and ATPase activity (Figure 5). In addition, in our study, the substrate (Mg^2+^-ATP) and Zn^2+^ were likely to have competed for the same ATP-hydrolysis site, indicating the binding of Zn^2+^ to the active site. This finding aligns with the data showing that Zn^2+^ can competitively bind to the catalytic center of various enzymes [22,53]. For example, Zn^2+^ at nanomolar concentrations inhibits the receptor protein tyrosine phosphatase β activity, which contains a catalytic cysteine residue [53].

Recently, three distinct Zn^2+^-binding sites on the GABA_A_R were identified: one site within the ion pore of the β3 subunit is for His267 and Glu270 residues, and the other two occur on the external amino (N)-terminal face between the β (Glu 182) and α (Glu137 and His141) subunits [10,11]. Early research questioned the possible role of a cysteine residue in the structure of pLGICs, which theoretically could interact with Zn^2+^ [8]. However, the involvement of cysteine in the Zn^2+^-inhibition potency of GABA_A_Rs was not demonstrated. Here, NEM completely inhibits the Cl^−^ATPase activity and Zn^2+^ eliminates the NEM effect on Cl^−^, HCO_3_^−^ATPase form (Figure 1), implicating it as the catalytic cysteine (Cys313) and nearby residues in the coordination of Zn^2+^ in the M3 domain of these β subunits (Figure 4F). This line of reasoning confirms that mutant isoforms do not show the activation of ATPase by NEM. In addition, the NEM effect on the GABA_A_R-mediated Cl^−^ or HCO_3_^−^ fluxes was eliminated by vanadate in the presence of HCO_3_^–^, which denoted a close site of localization of the ATP-hydrolyzing center and cysteine residue (C313) in the M3 domain of the β3 subunit [19]. However, it should be noted that a side chain of Cys313 faces inside the β-subunit and is buried between the TM domains of the surrounding residues making it difficult to access. Moreover, this amino residue is not a part of the channel and does not ensure its formation. Therefore, we can assume that NEM may cause not a direct, but an allosteric effect on the enzyme activity that does not consider cysteine residue as absolutely catalytic. Based on the data obtained, two possible molecular mechanisms for Zn^2+^ potency can be suggested: (1) the reaction of sulfhydryl bonds in the receptor-channel protein with Zn^2+^, and (2) the formation of inactive complexes between Zn^2+^ and the ATPase (Figure 5). Given that the ATP-hydrolyzing site is localized intracellularly and Cys313 is located in the transmembrane domain approximately in the middle of the membrane (Figure 4G), it is more likely that not only cysteine, but also other amino acid residues that form at the active center are also involved in the zinc-induced inhibition of enzyme activity.

Intracellular neuronal zinc modulation is associated with a variety of physiological signaling pathways (including protein kinases and protein phosphatases) [54,55,56]. Here, we expand on these data and demonstrate that, during brain maturation, Zn^2+^ with various efficacies inhibited not only the passive GABA_A_R-mediated responses, but also the ATPase compartment determined by β1 or β3 subunits. In addition, we established that the Cl^−^ATPase form belongs to the β1 subunit, whereas the β3 subunit preferably possesses the Cl^−^, HCO_3_^−^ATPase activity. Overall, we describe a new a role for Zn^2+^ in the inhibition of GABA_A_R-coupled ATPase activity and present evidence of the existence of a new intracellular site responsible for its potency via binding with cysteine. In this context, given the current structural and kinetic data, identifying the molecular determinants underlying the extracellular regulation of GABA_A_R function, intracellular Zn^2+^ regulation can have physiological and pathophysiological implications [57]. Indeed, GABAergic signaling is unique in that its polarity of action depends on [Cl^−^]_i_ and [HCO_3_^−^]_i_ [2,3], which are highly labile, leading not only to inhibitory, but also depolarizing/excitatory actions under certain conditions (for example, massive activation and spinal cord lesions, etc.) [35,57]. Moreover, altering [Cl^−^]_i_ on the second scale through changing GABA_A_R desensitization/resensitization may cause the collapse of the anion gradients and contribute to the induction of pathological conditions (e.g., seizures or epilepsy) modulated by Zn^2+^ [15,58]. A mechanistic understanding of the interactions between Zn^2+^ and the ATP-hydrolysis center within the receptor molecule may have clinical implications for the therapy of brain disorders by regulating the formation of an unstable, high-energy, phosphorylated intermediate and could pave the way for novel drug design.

## 4. Materials and Methods

### 4.1. Animals

Animal experiments were carried out using adult male Wistar rats purchased from the Institute of General Pathology and Pathophysiology vivarium and weighing 130–160 g at the time of arrival unless otherwise stated. Rats were always group-housed (5 per cage) and maintained in a temperature-controlled environment (23 ± 1) on a 12:12 h light-dark cycle and had access to food and water ad libitum. We performed all manipulations on animals in accordance with EU directive 2010/63/EU and according to the principles expressed in the Declaration of Helsinki revised by WMA, Fortaleza, Brazil, 2013, and the Rules of Good Laboratory Practice in the Russian Federation approved by Order N 199_H_ (1 April 2016) of the Ministry of Health Care, under supervision of the Ethics Committee of the Institute of General Pathology and Pathophysiology (project approval protocol No 3 of 18 August 2021; the final approval protocol No 1 of 3 March 2022).

### 4.2. Synaptoneurosomes and Plasma Membrane Preparation

SNs were prepared from whole brains of wild-type from freshly dissected forebrains (cortex) (~200–400 mg wet weight) as previously described [19]. Briefly, rats were quickly decapitated using a guillotine, brains were removed and placed in an ice-cold, balanced salt solution (BSS) containing 135 mM NaCl (sodium chloride, 1 mM KCl, 0.8 mM MgCl_2,_ 0.5 mM KH_2_PO_4_, 10 mM glucose, 0.1% bovine serum albumin (BSA), 10 mM Hepes-Tris (pH 7.3), and a protease inhibitor (A32955, Thermo Fisher Scientific, Waltham, MA, USA). The brain was cut into small pieces (2–3 mm) and manually homogenized (6 strokes) with a loosely fitting glass-Teflon homogenizer. The homogenate was passed through a nylon mesh (80 μm), and the filtrate was subsequently passed through a cellulose nitrate filter (8 μm) followed by centrifugation at 1000× *g* for 15 min. The pellet was washed once in BSS and centrifuged. All the procedures were performed at 4 °C. Sodium chloride (7647-14-5), potassium chloride (7447-40-7), magnesium chloride (7786-30-3), potassium phosphate monobasic (7778-77-0), BSA (9048-46-8), and d-(+)-Glucose (50-99-7) were obtained from Merck, Kenilworth, NJ, USA.

PMs were prepared from control HEK 293FT cells and various GABA_A_R variants were detached using Hanks’ balanced salt solution (Gibco, Waltham, MA, USA) without divalent cations (i.e., trypsin was not used), and the cells were centrifuged at 300× *g* for 3 min. The HEK 293FT cells or brain (mostly cortex) were homogenized in an ice-cold buffer containing 0.3 M sucrose, 0.5 mM EDTA-Tris, HEPES-Tris, 10 mM (pH 7.3), and protease inhibitor cocktail tablets (A32955, Thermo Fisher Scientific, Waltham, MA, USA), and centrifuged at 10,000× *g* for 15 min at 4 °C, after which the pellet was discarded. The supernatant was centrifuged for 1 h at 150,000× *g* and the resulting pellets were resuspended in 20 mM HEPES-Tris pH 7.3. This plasma membrane-enriched preparation was used for further measurements of the enzyme activity. Ethylenediaminetetraacetic acid (60-00-4), 4-(2-hydroxyethyl)-1-piperazineethane-sulfonic acid (HEPES), and Tris(hydroxymethylamino-methane (77-86-1) were obtained from Merck (Kenilworth, NJ, USA).

### 4.3. Cell Cultures and Transfection

For the expression homo- or heteromeric GABA_A_R ensembles, human embryonic kidney 293FT cells (American Type Culture Collection) were used. The cells were purchased from Invitrogen (Carlsbad, CA, USA) as part of the MembraneProTM Functional Protein Expression System (A11667), and the cell line identity was not further authenticated. The cells were grown and maintained in an incubator (Sanyo, Osaka, Japan) at 37 °C in a humidified atmosphere with 5% CO_2_, in DMEM media (41965-039, Gibco, Inchinnan**,** UK) supplemented with of 0.1 mM MEM NEAA (11140035, Gibco, Inchinnan, UK), 4 mM l-glutamine, 1 mM sodium pyruvate, 4.5 g/L d-glucose (15023021), and 10% FBS (10270-106, Gibco, Waltham, MA, USA) until the 20th passage, as suggested by the vendor. HEK 293FT cells were transfected by Lipofectamine TM 2000 or 3000 (Invitrogen, Thermo Fisher Scientific, Waltham, MA, USA and Lithuania) transfection reagents according to the manufacturer’s instructions. Cells were harvested and analyzed 24 h after transfection. For transfection procedures and virus-like particle (VLP) production, the same growth medium with decreased FBS content up to 4% was used according to the manufacturer’s recommendations. Geneticin G418 sulphate (11811031, Invitrogen, Waltham, MA, USA) was present in the growth medium at a concentration of 500 mg/mL constantly except during the transfection. The cells were subcultured at confluence by treatment with 0.05% trypsin and 0.02% EDTA in PBS. For selection purposes and improving the yield of VLPs, the transfection medium was removed after 24 h and a fresh growth medium with 10 µg/mL blasticidin (R21001 Gibco, Waltham, MA, USA) was added. Transfected cells and VLPs were collected and analyzed 24–48 h after transfection.

### 4.4. Molecular Biology

The genes encoding the full-length rat GABA_A_R β1, β2 or β3 subunits were amplified by PCR from the cDNA library (Evrogen, Moscow, Russia) using gene-specific primers with Kozak sequence at the 5′ end of the forward primer based on “GenBank:NM_012956.1”, “GenBank:NM_012957.2” “GenBank:NM_017065.1” sequences. The PCR products were cloned into the pEF6/V5-His TOPO TA vector (K961020, Invitrogen, Waltham, MA, USA) separately and verified by DNA sequencing. Each vector was amplified using *E. coli* TOP10 strain in LB medium supplemented with 20 µg/mL ampicillin. Isolation and purification of plasmids were performed with PureYieldTM Plasmid Miniprep System (Promega, Madison, WI, USA) and Plasmid Midiprep 2.0 (Evrogen, Moscow, Russia). The sterilization of plasmids was implemented via 0.22-µm filtration. The concentration of plasmids was evaluated on spectrophotometer NanoDrop 1000 (Thermo Fisher Scientific, Waltham, MA, USA). The quality validation of cloning and growth was performed additionally through enzymatic restriction by XbaI and BamHI in BamHI buffer (Thermo Fisher Scientific, Waltham, MA, USA), and the following electrophoresis in 1% agarose gel.

The typical transfection procedure of GABA_A_R subunit-containing constructs for the subsequent biochemical, spectrofluorometric, and Western blot analyses was as follows. Approximately 5 × 105 HEK293FT cells were suspended in 8 mL DMEM, plated into a 90-mm culture dish, and maintained 24 h approximately until 50–90% confluence. Then, 5 μg of plasmid DNA (β3 alone) was added combined with Lipofectamine^®^ 3000 Reagent (Invitrogen, Thermo Fisher Scientific, Waltham, MA, USA) in Opti-MEM^®^ I (1×) + GlutaMAXTM-I medium (51985-026, Gibco, Inchinnan, UK) accordingly the manufacturers’ recommendations. For microscopy, the cells were plated in 35 mm dishes and were incubated with a proportional amount of reagents and vectors.

For VLP production, GABA_A_R subunit-containing constructs were transfected together with Membrane Pro TM Reagent (Invitrogen, Waltham, MA, USA) amenably. Transfected HEK293FT started to bud off VLPs from the cell membrane approximately 24 h after transfection. The harvesting procedure was executed in conformity with manufacturer’s recommendations. Briefly, the VLP-containing medium was mixed with Membrane Pro^TM^ Precipitation Mix in the ratio of 5 to 1, where 5 refers to the medium. Then, the mix was incubated at 4 °C overnight. After incubation, VLPs were pelleted by centrifugation at 5500× *g* for 30 min and resuspended in HEPES buffer for subsequent analysis or stored at −80 °C.

### 4.5. Cl^−^ and HCO_3_^−^-Transport Monitoring

Cl^−^-sensitive fluorescent dye MQAE (N-(Ethoxycarbonyl-methyl)-6-Methoxyquinolinium Bromide) or pH-sensitive fluorescent dye BCECF, AM (2′,7′-Bis-(2-Carboxyethyl)-5-(and-6)-Carboxyfluorescein, Acetoxy methyl Ester) were obtained from Thermo Fisher Scientific, USA (E3101) or B1170, respectively. A stock solution was prepared in H_2_O, aliquoted, and stored in the freezer (−20 °C) protected from light. For cases in which fluorescence measurements were conducted, the HEK 293FT cells or SNs with loaded dye were stored in an opaque test tube at RT or 4 °C. Sodium bicarbonate (144-55-8), γ-aminobutyric acid, GABA (56-12-2), ouabain, bicuculline (485-49-4) and zinc chloride (7646-85-7) were obtained from Sigma-Aldrich (St. Louis, MI, USA), and bumetanide (28395-03-1) were obtained from Merck (Kenilworth, NJ, USA). SNs were loaded dye (MQAE or BCECF) in BSS for 1 h at 37 °C and stored in an opaque test tube at RT or 4 °C. For that, the control HEK 293FT cells and various GABA_A_R β3 isoforms cells were trypsinized by adding 0.05% trypsin-EDTA solution (25200056, Gibco BRL, Waltham, MA, USA), washed PBS twice, resuspended in the BSB, and then loaded with MQAE for 1 h at 37 °C. After loading, the suspension was centrifuged at 200× *g* for 5 min at RT and kept in the aforementioned medium at RT in the opaque test tubes. For analysis, the pellet was resuspended in the BSB. Monitoring was performed with cells continuously superfused with incubation medium composed of (mM):135 NaCl (or 135 NaCl and 25 NaHCO_3_), 0.5 KH_2_PO_4_, 0.8 MgCl_2_, and 5 mM Hepes (pH 7.4). Dye-loaded cells (SNS or HEK 293FT) were equilibrated in the test tube in the incubation medium V = 200 µL) in the absence or presence of compounds (ZnCl_2_, Na_3_VO_4_, NEM, bicuculline, or ouabain) for about 10 min at 37 °C before initial fluorescence measurements, and then 150 μL of the suspension was added into quartz microcuvette (non-flow cell) and stirred. The GABA-mediated Cl^−^ or HCO_3_^−^ transport was initiated directly by addition of GABA in final concentration of 1–100 µM in the cuvette by an in-house solution supply system. GABA_A_R-mediated Cl^—^or HCO_3_^—^transport was assessed by the dynamic measurements of the variations in the fluorescence intensity of Cl^—^sensitive fluorescent dye MQAE-loaded or BCECF-loaded HEK 293FT cells or SNs using a FluoroMax^®^-4 spectrofluorometer (HORIBA Scientific Edison, Piscataway, NJ, USA), respectively. The excitation and emission wavelengths were 350 nm and 480 nm for the measurement of Cl^−^-transport or 490 nm and 535 nm for measurement of HCO_3_^−^-transport, respectively. The Δ*F*/*F* of each trial was calculated as (*F* − *F*_0_)/*F*_0_, where *F*_0_ is the baseline fluorescence signal averaged over a 10 s period (this was the control measurement) immediately before the start of the application of GABA and supplement compounds. The value of 100% was obtained as the fluorescence intensity before the application of GABA, in the absence or presence of test compounds. The maximum amplitude of GABA-mediated fluorescence responses was calculated as the maximal difference in fluorescence intensity in the absence or presence of an agonist.

### 4.6. ATPase Activity Monitoring

The ATPase activity in PMs of neurons or HEK 293FT cells expressing the various constructs were measured as previously described [20]. PMs (~7 µg) or VLPs (~14 µg) were added into glass tube to 0.5 mL of an incubation medium containing 20 mM HEPES-Tris pH 7.3, 5 mM NaCl/25 mM NaHCO_3_ (or 25 mM NaCl), 0.5 mM ouabain and 40 mM NaNO_3_ (neutral salt) to measure enzyme activity. The PMs or VLPs were preincubated at 37 °C for ~20 min with the relevant compounds (NEM (0.1–400 µM); Zn^2+^(0.001–100 µM); DTT (2 mM); bicuculline (40 μM)) in the incubation medium. Preparation of the test tube with bicarbonate—NaHCO_3_ (1 mM)—was previously dissolved in HEPES (20 mM) and then added in the 20 mM HEPES-Tris buffer (pH 6.7). After preincubation, the enzyme reaction was started by addition of Mg^2+^-ATP 2 mM (final concentration) in the incubation medium.

After 15–20 min of incubation, the ATPase activity was stopped by the addition of reagents for measuring of inorganic phosphorus (P_i_). The Cl^−^- and Cl^−^, HCO_3_^−^ATPase activities were determined as a difference in formation of P_i_ in the absence and in the presence of NaCl (2–60 mM) or NaHCO_3_ (2.5–25 mM) in the incubation medium, respectively. Adenosine 5′-triphosphate (ATP) disodium salt hydrate (34369-07-8) and adenosine 5′-triphosphate disodium salt hydrate (A26209) were obtained from Merck (Kenilworth, NJ, USA). The concentration of P_i_ in the incubation medium was measured by a modified method of Chen et al., (1956) [19] using a Cary 60 UV–vis spectrophotometer (Agilent, Santa Clara, CA, USA) at wavelength of 650 nm. The γ-phosphate analog, orthovanadate (VO_4_^3−^) (Sigma-Aldrich, St. Louis, MI, USA), was obtained by boiling the vanadate solution (pH 10; 10 min), and freshly boiled stock was diluted to the final concentration (pH 7.3) prior to use.

### 4.7. Western Blot

VLPs of transfected HEK 293FT cells were subjected to SDS-PAGE using the SDS-PAGE reagent starter kit (1615100 Bio-Rad, Hercules, CA, USA) and to Western blot analysis using the Pierce™ fast Western blot kit (35055 Thermo Scientific, Waltham, MA, USA), ECL Plus Western Blotting Detection System Substrate (GE Healthcare, Chicago, IL, USA). Samples were SDS-treated by boiling for 5 min in a buffer consisting of 62.5 mM Tris, 10% glycerol, 5% 2-mercaptoethanol, 4% SDS, and 0.001% bromophenol blue, and then ~20 µg of total protein was loaded into SDS-PAGE. Electrophoresis parameters were: 70 V for 10 min on 4% SDS-PAGE stacking gel and 120 V for 50 min on 12.5% SDS-PAGE resolving gel. Proteins were transferred on PVDF membrane by the semi-dry method using 0.09 A/cm^2^ for 1 h. After that, membranes were incubated for 1 h in blocking solution containing 5% milk, and then incubated at 4 °C overnight with primary Anti-GABRB1 Monoclonal Antibody (S96-55) (Catalog # MA5-27699, TermoFisher Scientific, Waltham, MA, USA), Anti-GABRB2 Recombinant Rabbit Monoclonal Antibody (ARC0631) or GABRB3 antibody [N87/25] (ab98968, Abcam, Cambridge, UK), diluted 1:1000 with the blocking solution. After incubation, the membranes were washed with TBS-T 4 times for 15 min each, and then incubated at RT for 1 h with secondary HRP-conjugated antibodies (62–6520 Thermo Fisher Scientific, Waltham, MA, USA) diluted 1:5000 with the blocking solution. Then, the membrane was washed with TBS-T four times and the GE Healthcare ECL Plus Western Blotting Detection System (Amersham^TM^, GE Healthcare, UK) was applied according to manufacturer’s instructions. The visualization of the bands was performed using a Kodak Image Station 440 (Rochester, NY, USA).

### 4.8. Statistical Analysis

The data are expressed as the mean ± SEM, and differences were considered significant for *p* < 0.05. Statistical differences were determined by two-tailed Student’s unpaired *t*-test for data with equal variances and which were assessed as normally distributed with the Shapiro–Wilk test. Graphs and statistical analysis were obtained by using GraphPad Prism 9.3 (GraphPad Software, San Diego, CA, USA).

Representative images of MQAE and BCECF fluorescence changes in synaptoneurosomes VLPs were analyzed and assessed using Origin Pro version 9.1 for Windows (OriginLab, Northampton, MA, USA). The mean fluorescence intensity from each treatment group was separately compared to the mean fluorescence intensity of the untreated control group.

## Data Availability

The data presented in this study are available on request from the corresponding author.
